# Development and validation of a novel patient-reported outcome for microscopic colitis—Microscopic Colitis Score (MCS)

**DOI:** 10.1093/ecco-jcc/jjaf153

**Published:** 2025-10-13

**Authors:** Katarina Pihl Lesnovska, Samuel Schäfer, Yamile Zabana, Ingrid Fajardo Anes, Danila Guagnozzi, Emese Mihaly, Stephan Miehlke, Ahmed Madisch, Beatrice Marinoni, Giovanni Latella, Andreas Münch, Henrik Hjortswang

**Affiliations:** Department of Gastroenterology and Hepatology, Linköping University Hospital, Linköping, Sweden; Department of Health, Medicine and Caring Sciences, Linköping University, Linköping, Sweden; Department of Gastroenterology and Hepatology, Linköping University Hospital, Linköping, Sweden; Department of Health, Medicine and Caring Sciences, Linköping University, Linköping, Sweden; Department of Biomedical and Clinical Sciences, Linköping University, Linköping, Sweden; Department of Gastroenterology, Hospital Universitari Mútua Terrassa, Terrassa, Spain; Centro de Investigación Biomédica en Red en Enfermedades Hepáticas y Digestivas, Madrid, Spain; Department of Gastroenterology, Hospital Universitari Mútua Terrassa, Terrassa, Spain; Centro de Investigación Biomédica en Red en Enfermedades Hepáticas y Digestivas, Madrid, Spain; Department of Gastroenterology, University Hospital Vall de Hebron, Barcelona, Spain; Department of Internal Medicine, Semmelweis University, Budapest, Hungary; Center for Digestive Diseases, Internal Medicine Center, Hamburg, Germany; Center of Internal Medicine, Hospital DIAKOVERE Friederikenstift, Hannover, Germany; Gastroenterology and Endoscopy Unit, Fondazione IRCCS Ca’ Granda Ospedale Maggiore Policlinico, University of Milan, Milan, Italy; Gastroenterology, Hepatology and Nutrition Division, Department of Life, Health and Environmental Sciences, University of L’Aquila, L’Aquila, Italy; Department of Gastroenterology and Hepatology, Linköping University Hospital, Linköping, Sweden; Department of Health, Medicine and Caring Sciences, Linköping University, Linköping, Sweden; Department of Gastroenterology and Hepatology, Linköping University Hospital, Linköping, Sweden; Department of Health, Medicine and Caring Sciences, Linköping University, Linköping, Sweden

**Keywords:** microscopic colitis, patient reported outcome measures, microscopic colitis score

## Abstract

**Background & Aims:**

Despite debilitating symptoms, no standardized disease severity index exists for microscopic colitis (MC). This gap hinders alignment with U.S. Food and Drug Administration (FDA) and European Medicines Agency (EMA) standards, which emphasize the importance of patient-reported outcome measures (PROMs) in new therapy approval. This study aimed to validate the Microscopic Colitis Symptom Questionnaire (MCSQ) and develop the Microscopic Colitis Score (MCS), a novel disease severity index.

**Method:**

This prospective, multicenter study included 131 patients with biopsy-confirmed MC (67 remission, 64 active disease). Patients completed MCSQ and health-related quality of life (HRQoL) assessments [IBDQ-32, Short Health Scale (SHS)] at baseline and follow-up. Clustering analysis systematically identified distinct disease severity groups. MCS was developed as a composite score derived from MCSQ.

**Results:**

Factor analysis revealed a three-factor MCSQ model with good internal consistency (Cronbach’s alpha = 0.88). Test–retest reliability (intraclass correlation coefficient = 0.88) and responsiveness to treatment (*P* < .01) of all MCSQ items were high. MCS, ranging from 0 (asymptomatic) to 15 (maximum symptoms), correlated strongly with HRQoL measures such as IBDQ-32 total score (*r*_p_=−0.78), IBDQ-32 bowel symptoms (*r*_p_=−0.80), and SHS bowel symptoms (*r*_p_=0.69). Receiver–operating characteristic curves indicated that MCS could accurately identify patients in remission [as per Hjortswang criteria; area under the curve (AUC) = 0.85], as well as mild (AUC = 0.97), moderate (AUC = 0.93), or severe disease (AUC = 0.96).

**Conclusions:**

MCSQ and MCS are valid, reliable, and responsive tools that meet FDA and EMA standards. Both accurately reflect the diverse symptoms of MC. Compared to the binary Hjortswang criteria, MCS provides a nuanced evaluation of disease activity and holds promise for assessing therapeutic efficacy in future trials.

## 1. Introduction

Microscopic colitis (MC), encompassing collagenous colitis (CC) and lymphocytic colitis (LC), is a chronic inflammatory bowel disease predominantly affecting elderly women.^1^ Although its etiology remains unclear, the incidence of MC is rising and is currently estimated to be 11.4 (9.2–13.6) cases per 100 000 person-years.^2^ Hallmark symptoms include chronic watery diarrhea, urgency, fecal incontinence, and nocturnal stools.^2–4^ Despite normal macroscopic findings during endoscopy, MC is characterized by distinct histological features of chronic mucosal inflammation.^1^^,^^5^ While MC does not significantly increase mortality or lead to severe complications, active disease can severely impair patients’ health-related quality of life (HRQoL).^2^^,^^5–8^ The primary therapeutic goal is therefore to restore bowel function and improve HRQoL.^6^^,^^9^ Yet, treatment options are limited, with budesonide being the only moderately evidence-based treatment option,^2^^,^^10–12^ often followed by symptom recurrence upon cessation.^11^ Off-label use of biologics such as adalimumab, infliximab, and vedolizumab for steroid-refractory MC has produced exiting results, but prospective trials with standardized outcomes are needed.^13^

Despite the microscopic finding of chronic inflammation, there is still no reliable biomarker to assess disease activity in MC.^2^^,^^5^ Instead, assessment of MC disease activity currently relies on the Hjortswang criteria (≥3 stools/day or ≥1 watery stool/day).^2^^,^^9^ While these criteria are widely used,^2^^,^^6^^,^^14–16^ they lack the granularity to capture the full spectrum of symptoms or differentiate disease severity levels. Moreover, they were developed specifically for CC and lack validation for LC.^9^ An early attempt to address this gap led to the development of the Microscopic Colitis Disease Activity Index (MCDAI),^3^ modeled after the Crohn’s Disease Activity Index (CDAI).^17^ MCDAI integrates multiple symptoms, including stool frequency, nocturnal stools, abdominal pain, weight loss, urgency, and incontinence, into a weighted formula. However, MCDAI is limited by (1) reliance on physician global assessment of a single investigator for validation; (2) poor responsiveness due to long-term metrics (eg, weight loss and number of monthly fecal incontinence episodes), which may not capture rapid symptom changes typical of MC exacerbations or responses to treatment; (3) the complexity of calculating this score in a clinical setting; and (4) the lack of reference intervals for MCDAI which complicate interpretation.

Additionally, neither MCDAI nor the Hjortswang criteria have been subject to formal, prospective validation nor do they meet the latest regulatory requirements from the U.S. Food and Drug Administration (FDA) for patient-reported outcomes (PROs) in clinical trials.^2^^,^^18^ This is particularly important as the FDA and European Medicines Agency (EMA) have recently emphasized the importance of robust clinical outcome assessments, particularly PROs, in regulatory decision-making.^19^^,^^20^ To address the need for a responsive scoring tool for MC that not only aligns with these regulatory standards but also accurately captures the diverse symptoms of the disease, we have recently developed the six-item Microscopic Colitis Symptom Questionnaire (MCSQ; initially called E-MCAI).^21^ In this study, we aimed to evaluate MCSQ validity and responsiveness and to address the remaining gaps by developing and validating the Microscopic Colitis Score (MCS). The MCS is designed to assess MC severity comprehensively, aligning with regulatory requirements while capturing the diverse symptoms of MC. We find that MCS reliably predicts patient-reported HRQoL as measured by IBDQ-32 and Short Health Scale.^22–24^

## 2. Materials and methods

### 2.1. Study design

This prospective multicenter study characterized symptomology and HRQoL in patients with MC. Patients were included from six European gastroenterological centers: Linköping University Hospital, Sweden; University Hospital Vall de Hebron-Barcelona, Spain; Hospital Universitari Mútua de Terrassa Universitat de Barcelona, Spain; Semmelweis University, Hungary; and KRH Clinic Siloah, Germany; Fondazione IRCCS Ca’ Granda Ospedale Maggiore Policlinico Milano, Italy. The study was conducted according to the principles of the *Declaration of Helsinki*, International Council for Harmonisation Good Clinical Practice guidance, and was approved by The Medical Research Ethics Committee of Linköping Dnr 2019-02760 and by the institutional review boards of each participating center. All patients gave written informed consent before inclusion.

All patients filled out questionnaires at baseline and follow-up (Table 1). If patients sought health care due to active disease, they were asked to fill in MCSQ retrospectively. Disease activity was defined as mean daily stools ≥3 or mean daily watery stools (Bristol 7) ≥1, according to the Hjortswang criteria.^9^ Patients with active disease (as per Hjortswang criteria) were recruited during out-patient contacts for active disease and were treated with oral budesonide capsules 3 mg three times daily for 4 weeks after completion of baseline questionnaires. Patients in remission were recruited by postal survey. We aimed to include remission and active disease patients approximately 1:1 to evaluate validity, reliability, and responsiveness optimally.

**Table 1.** Questionnaires for all study groups and timepoints.

Questionnaire	Baseline (all patients)	**2** **weeks (remission at baseline)**	**4** **weeks (active disease at baseline)**
Disease history	X		
Demographics	X		
Rome IV criteria	X		
Disease course	X		
MCSQ	X	X	X
SHS	X	X	X
IBDQ-32	X	X	X
Health transition	X	X	X

### 2.2. Patient and public involvement

Patients were not involved in the development of the research question and outcome measures apart from participation in cognitive interviews for cross-cultural adaptation of the instrument. However, the development of MCSQ was based on substantial patient input, including the identification and prioritization of relevant symptoms in comparison to expert assessments, as detailed in the original development publication.^21^

### 2.3. MCSQ design

The MCSQ development followed FDA and EMA guidelines for the development of PROs.^19^^,^^20^ In summary, this development included a literature review, qualitative interviews symptom ranking by MC experts, a symptom survey (79 MC patients, 70% response rate), and following expert review and cognitive interviews before MCSQ was piloted in 100 MC patients (66% response rate).^21^ MCSQ assesses bowel symptom burden daily over 7 days and records total daily stools, nocturnal stools (disrupting sleep), solid and loose stools (corresponding to Bristol 6 and 7), urgency, leakage, and abdominal pain.^21^^,^^25^ Questions have high scale-level content validity indices (S-CVI universal agreement UA = 0.99, S-CVI average = 0.99 for relevance; S-CVI universal agreement = 0.82, S-CVI average = 0.97 for clarity and simplicity).^21^

MCSQ was originally developed in Swedish; an English ­version was produced through forward–backward translation, with discrepancies resolved by consensus. Cognitive interviews were subsequently conducted with patients to ensure cultural validity and clarity. From the English version, translations into Hungarian, Italian, Spanish, and German were performed using a standardized forward–backward translation procedure.^26^ In each language, cognitive debriefing interviews with patients assessed comprehension, relevance, and cultural applicability, ensuring the conceptual equivalence of the instrument across different cultural contexts. During such interviews, participants were asked to complete the questionnaire while verbalizing their thought processes (“think-aloud” technique).^27^ Additionally, targeted probing questions were used to explore the patients’ understanding of the items, the relevance of the questions to their experience, and any potential cultural or linguistic ambiguities. Interviews were audio-recorded and analyzed to identify problems related to comprehension, retrieval, judgment, and response processes. Necessary revisions were made to ensure clarity, conceptual equivalence, and cultural appropriateness across all language versions.

#### 2.3.1. Inclusion/exclusion criteria

Participants were 18 years or older at the time of inclusion. Only individuals with a biopsy-verified diagnosis of MC were included. If the MC subtype was specified, histological findings had to be consistent with diagnostic criteria for the respective subtype (LC or CC).^1^ Clinical symptoms compatible with MC at the time of diagnosis also had to be present. Ongoing treatment with budesonide at the time of inclusion led to exclusion.

### 2.4. Other questionnaires

Patients completed the Inflammatory Bowel Disease Questionnaire (IBDQ), a 32-item assessment designed for classic IBD but with previously proven relevance to MC.^3^^,^^9^ Responses are rated on a seven-point Likert scale. Items can be divided into four domains: bowel symptoms, systemic symptoms, social function, and emotional function. Total scores range from 32 to 224, with higher scores indicating better HRQoL. HRQoL was also assessed using an adapted version of the Short Health Scale (SHS), which uses non-leading, open-ended questions to assess four key dimensions of subjective health (bowel symptoms, social function/interference with daily life, disease-related worry, and general well-being).^24^ Responses were recorded on a six-point Likert scale. Patient perception of the disease course was assessed using curves from the IBSEN Study.^28^ Irritable bowel syndrome (IBS) symptoms were evaluated using a questionnaire for the Rome IV criteria.^29^ Patient-perceived severity of symptoms (none, mild, moderate, severe symptoms) and the transition of symptoms compared to 2 weeks ago (much better, better, same, worse, much worse) was assessed through the Health Transition Questionnaire.^28^

### 2.5. Statistics

Statistics were conducted in R v.4.4.1. Depending on the distribution of variables, they are either reported as (1) mean and SD, (2) median and IQR, or (3) number and percentage. Depending on data type and distribution, suitable statistical tests were used and are indicated in each instance. Bonferroni correction was applied when multiple comparisons occurred.

#### 2.5.1. Validity

Exploratory factor analysis (EFA) was conducted using the *psych* R package (v.2.4.6.26)^30^ to examine MCSQ’s theoretical structure. Data eligibility for factor analysis was confirmed using Bartlett’s test of sphericity (*P* < .05) and Kaiser–Meyer–Olkin measure of sampling adequacy (KMO ≥ 0.6). Cronbach’s alpha assessed variance and covariance among MCSQ items to evaluate internal consistency. Furthermore, validity was evaluated by testing a set of hypotheses about expected correlations between the MCSQ and HRQoL instruments, such as IBDQ-32 and SHS, as well as patient experience. We expected a moderate correlation (Pearson *r*_p_ ≥ 0.4) for most MCSQ items.

#### 2.5.2. Reliability

Test–retest reliability of the questionnaire was assessed using the intraclass correlation coefficient (ICC; *psych* R package), employing a two-way mixed-effects model with absolute agreement (ICC2k).^31^^,^^32^ Test–retest reliability was calculated using only subsets of participants who had completed the questionnaire twice, with 2 weeks between administrations. ICC > 0.75 indicates good reliability.

#### 2.5.3. Responsiveness

As MCSQ could ideally be used to evaluate potential treatment effects, it was critical that values reflect the dynamic changes upon treatment-related improvement in patients with active disease at baseline. For this, a paired Wilcoxon signed-rank test was performed for each item, comparing paired measurements between baseline and follow-up.

#### 2.5.4. Clustering

Clustering requires complete data. Hence, we removed patients with >20% missing data points at a given timepoint (baseline or follow-up; 42 patient–timepoint combinations removed), before variables that had ≤20% missing data points were removed (one variable; ratio of fluffy to watery stool). Next, the remaining missing data points (3.3%) were imputed using non-parametric missing value random forest imputation (*missForest* R package). Data were then scaled and underwent principal component analysis (PCA) and JackStraw calculation (six relevant dimensions) for PCA. Clustering analysis was performed using the Seurat v.5.1 package.^33^ A shared nearest-neighbor graph was constructed, and neighborhood overlap between every cell and its *k*-nearest-neighbors (*k* = 10) was calculated based on the Jaccard index. Next, clusters were identified by applying the Leiden algorithm to the shared nearest-neighbor graph with a resolution of 0.9. Clusters were visualized using UMAP.

#### 2.5.5. Microscopic Colitis Score

The association between all diarrhea-related MCSQ items and HRQoL was assessed to create a disease-activity score. Previous studies had indicated that a clinically significant difference corresponds to ca. 20 IBDQ-32 points.^34^ We therefore set cut-offs and scored MCSQ items so that any point corresponded to 15–20 IBDQ points. The MCS is then derived by addition of item-specific points. This scoring approach is similar to other prominent scores, such as the Child–Pugh Score for cirrhosis mortality.^35^^,^^36^

Validity, reliability, responsiveness, and relevance were evaluated for MCS, similar to MCSQ. The optimal MCS cut-offs for disease severity classification were assessed by receiver operating characteristic (ROC) curves, and the total area under the curve (AUC) was calculated. Sensitivity and specificity for the chosen cutoffs are presented.

## 3. Results

Initially, 133 patients were enroled. However, two patients (1.5%) did not complete baseline questionnaires and were excluded. The completion rates of all administered questionnaires are reported in Figure S1. Most importantly, MCSQ was completed by all 131 patients (*n* = 67 remission, *n* = 64 active disease at baseline), and 114 patients responded to MCSQ at follow-up (*n* = 57 remission at baseline, including 15 partial completions; *n* = 57 active disease at baseline, including 13 partial completions).

Characteristics of the patients (68 CC, 59 LC, and four unspecified MC) are displayed in Table 2. No significant differences between CC and LC were found regarding demographics, clinical features, MCSQ item response, and HRQoL measures (IBDQ-32 total, SHS bowel symptoms, SHS social function). Therefore, the following analyses were performed on all patients jointly.

**Table 2.** Patient characteristics at baseline.

	Collagen colitis (*n* = 68)	Lymphocytic colitis (*n* = 59)	Total (*n* = 131)	*P*-value CC vs LC
Age, median (IQR) years	60 (53.5–75.0)	68 (53.5–74.5)	66 (53.5–74.0)	0.43 ^Wilc^
Disease duration, median (IQR) years	3.6 (0.2–5.7)	3.2 (1.1–5.7)	3.6 (0.6–5.9)	0.61 ^Wilc^
Female, *n* (%)	56 (82.4)	48 (81.4)	108 (82.4)	0.88 ^Chi2^
Active disease (Hjortswang criteria), *n* (%)	35 (51.5)	27 (45.8)	64 (48.9)	0.52 ^Chi2^
Marital status: Single, *n* (%)	20 (29.4)	14 (23.7)	34 (26.0)	0.52 ^Chi2^
Smoking, *n* (%)				0.08 ^Fish^
Never	28 (41.2)	33 (55.9)	63 (48.1)	
Used to	24 (35.3)	21 (35.6)	48 (35.1)	
Currently	15 (22.1)	5 (8.5)	21 (16.0)	
Country, *n* (%)				0.74 ^Fish^
Sweden	51 (75.0)	45 (76.3)	96 (73.3)	
Spain	8 (11.8)	6 (10.2)	14 (10.7)	
Hungary	3 (4.4)	5 (8.5)	12 (9.2)	
Germany	4 (5.9)	3 (5.1)	7 (5.3)	
Italy	2 (2.9)	0 (0.0)	2 (1.5)	
Irritable bowel syndrome (IBS), *n* (%)	9 (13.2)	9 (15.3)	20 (15.3)	0.74 ^Chi2^
Coexisting disease other than IBS, *n* (%)	56 (82.4)	50 (84.7)	109 (83.2)	0.99 ^Chi2^

Abbreviations: CC, collagenous colitis; LC, lymphocytic colitis; IBS, irritable bowel syndrome; IQR, interquartile range. ^Wilc^, Wilcoxon sign rank test; ^Chi2^, Chi^2^ test; ^Fish^, Fisher’s exact test.

### 3.1. Evaluation of MCSQ

**Figure 1.**
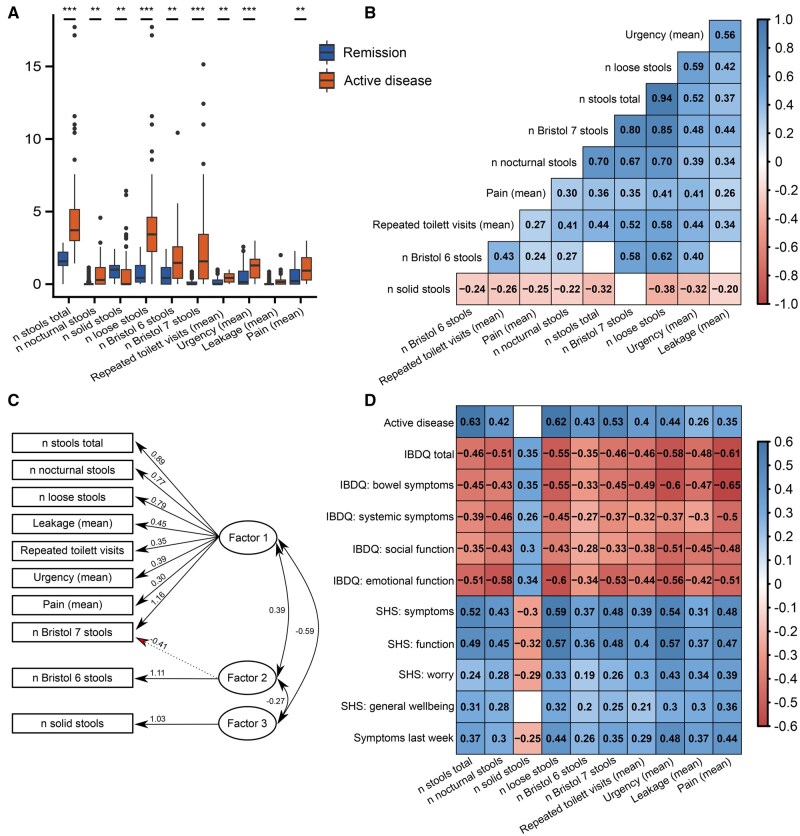
Evaluation of MCSQ validity. (A) Item response variation for MCSQ questionnaires from both timepoints (baseline *n* = 131; follow-up *n* = 109) by disease activity at respective timepoints (*n* = 145 remission, *n* = 95 active disease). (B) Pearson correlation coefficients for MCSQ inter-item correlations. (C) Path diagram representation of Promax rotated factor loadings (>|0.4|) and factor intercorrelation resulting from exploratory factor analysis (EFA). (D) Pearson coefficients for MCSQ item correlation with disease activity, HRQoL, and patient experience of microscopic colitis symptoms. Active disease (0 = remission, 1 = active disease) as per Hjortswang criteria. The six-point Likert scale for SHS ranged from 1 (best) to 6 (worst). Patients rated the occurrence of microscopic colitis symptoms during the previous week, ranging from 0 (none) to 3 (severe). In (B) and (D), non-significant correlations are shown as blanks. *P < .05; **P < .005; ***P < .0005; IBDQ, Infammatory Bowel Diseseas Questionaire; SHS, Short Health Scale.

Responses spanned the full range of response options for all ordinal variables (Figure 1A). The distribution of response levels differed significantly between patients with active disease and remission (as per Hjortswang criteria), as well as between patients before and after treatment. Furthermore, most items were well inter-correlated (|Pearson *r*| > 0.3) except for the mean number of solid stools (Figure 1B).

#### 3.1.1. Validity

The factorability of MCSQ items was explored using EFA. Eligibility of data for EFA was confirmed by Bartlett’s test of sphericity (*P* < 10^−16^) and KMO of 0.690, which is well above the minimum requirements for conducting EFA. We considered several criteria for deciding on the number of factors to extract. The criteria included the Kaiser–Guttman eigenvalue method, which suggested three factors, and parallel PCA, which suggested one factor. Hence, a three-factor model was derived and compared to a one-factor model. Item loading for the three-factor model ranged from 0.41 (mean pain) to 1.00 (mean *n* of loose stools) and was slightly superior to the one-factor model, where item loading ranged from |−0.37| (mean *n* solid stools) to 1.00 (mean *n* of loose stools). Lastly, factors in the three-factor model were Promax rotated, and factor intercorrelations remained high (Figure 1C). Eigenvalues and variance are presented in Table S1. Cronbach’s alpha (coefficient alpha reliability) was 0.886 for Factor 1 loadings (>|0.4|) and 0.825 for all items in MCSQ, suggesting good internal consistency.

Since we aimed to develop a tool that indirectly measures HRQoL, we evaluated to what extent MCSQ item responses were concurrent with dedicated HRQoL surveys such as the IBDQ-32 and SHS as well as patient experience (Figure 1D). We found that all items exhibited strong correlations (>|0.3|) to relevant measures.

#### 3.1.2. Reliability

**Figure 2.**
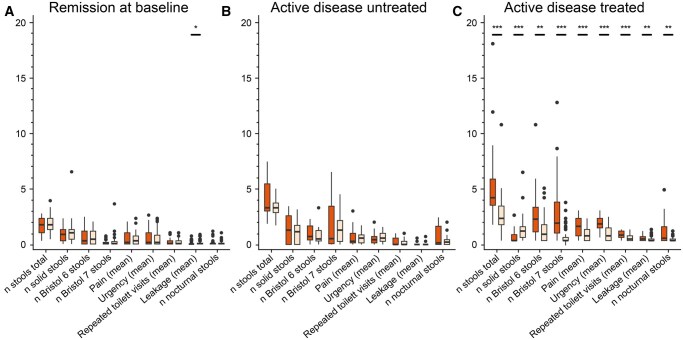
Reliability and responsiveness of MCSQ items based on disease activity. (A) Test–retest of MCSQ for patients in remission at baseline (*n* = 56). Colors represent baseline (orange) and follow-up (beige). (B) Patients with active disease at baseline who were not treated (*n* = 13), as no medical care was sought. (C) Patients with active disease at baseline who received treatment with corticosteroids (*n* = 45) showed improvement in all items at follow-up. Paired Wilcoxon signed-rank tests were performed for all scenarios between baseline and follow-up, and *P*-values were Bonferroni-corrected. *P < .05; **P < .005; ***P < .0005.

To assess the test–retest reliability of MCSQ, intraclass correlation coefficients were derived by comparing responses of patients between baseline and follow-up for patients who were in remission at both timepoints. Overall ICC (95% CI) for MCSQ was 0.88 (0.86–0.90), corresponding to excellent reliability. Paired analyses for all individual MCSQ items of patients who had been in remission (untreated) at both timepoints showed no significant differences between timepoints (Figure 2A). Similarly, we assessed ICC for 11 patients who have had active disease at baseline but received no treatment and still had active disease at follow-up 2 weeks later. Overall ICC was 0.87 (0.81–0.91). Again, paired comparison indicated no significant differences in item response between baseline and follow-up (Figure 2B), indicating good reliability. Note that these 11 patients had not received treatment for their active disease, as they had not sought care for their symptoms, but rather were discovered retrospectively in the postal survey that aimed to establish a control group.

#### 3.1.3. Responsiveness

Paired analysis of patients who had active disease at baseline and were treated showed a significant improvement in all MCSQ items (Figure 2C), which suggests the feasibility of using MCSQ in future drug trials. Only four patients progressed from remission at baseline to active disease at follow-up and hence no statistical comparison of MCSQ response could be made.

### 3.2. Cluster-based definition of disease severity groups

We aimed to develop a score for MC severity that ideally differentiates between patients in remission, mild, moderate, and severe disease. However, one major hurdle was that no previous definition for mild or severe disease existed. To date, the only established criteria for MC are the Hjortswang criteria.^9^ These criteria were developed to identify CC patients in need of treatment. While clinically immensely useful, such criteria fail to differentiate between, for example, moderate and severe disease. To establish disease severity groups for MC, we therefore started by characterizing our patient cohort to explore which patient groups naturally existed in our data.

**Figure 3.**
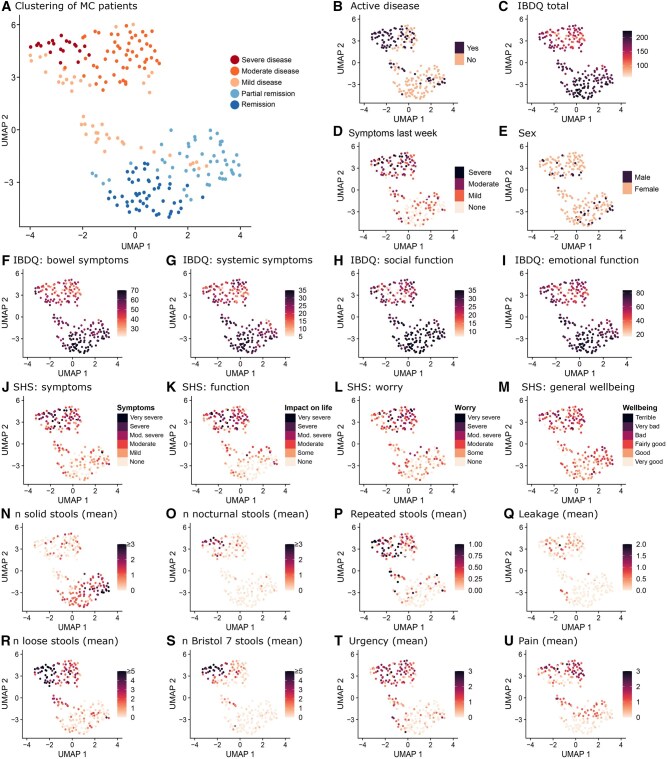
Clustering of microscopic colitis patients. Using all available information, patients with sufficient data points (*n* = 129 at baseline and *n* = 91 at follow-up) were clustered to identify patient subgroups. Clustering revealed five distinct patient subgroups. Three clusters identified patients with decreased quality of life and bowel symptoms that correspond to mild to severe disease, and two clusters that corresponded to patients in remission. No sex bias was found in any cluster (E). Note that IBDQ-32 total ranges from 32 (worst) to 224 (best). IBDQ-32 total score is derived from four dimensional scores: bowel symptoms (10–70), systemic symptoms (5–35), social function (5–35), and emotional function (12–84). IBDQ, Inflammatory Bowel Disease Questionnaire; SHS, Short Health Scale.

In this regard, clustering is a natural choice, as it systematically groups patients with similar data points into homogeneous groups while maximizing heterogeneity across groups. At the same time, the Leiden clustering algorithm deployed by us does not rely on pre-defined assumptions about group characteristics, distribution, frequency, or an assumption about the number of formed clusters. Clustering of patient data (HRQoL, symptom diary, demographics) revealed the presence of five distinct patient groups (Figure 3). Clusters were annotated based on their distinct characteristics and could be categorized into remission, partial remission, as well as mild/moderate/severe disease.

### 3.3. Microscopic Colitis Score

**Figure 4.**
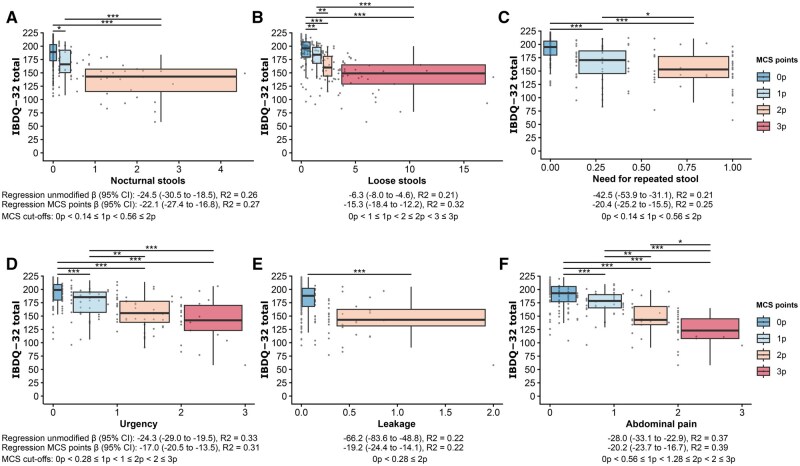
Cut-offs for awarding item-specific MCS points. All plots show an association of item-specific values to Inflammatory Bowel Disease Questionaire (IBDQ) total points for all 32 items. For each item, cut-offs were identified, and items were scored so that each item-specific MCS point corresponded to a 15–20 point decrease in IBDQ-32 total. Item-specific MCS points are indicated as boxplots; width corresponds to the cut-off interval, and color corresponds to MCS points. Under each plot, we indicate the outcome of univariate linear regression for IBDQ-32 total using (1) the unmodified item and (2) item-specific MCS points as well as numerical cut-offs. All univariate analyses were significant. Wilcoxon signed-rank tests were performed, and *P*-values were Bonferroni corrected. *P < .05; **P < .005; ***P < .0005.

To derive an easy-to-use and clinically relevant scoring system, we started by categorizing and scoring each MCSQ item to correspond to 15–20 IBDQ-32 points. While this point system facilitates easy calculation of MCS, cut-offs did not decrease the predictive performance of MCS variables (Figure 4). MCS was derived by summation of item-specific scores (Table S2). As the number (*n*) of loose stools is directly derived from two MCSQ items (*n* of Bristol 6 and *n* of Bristol 7 stools), univariate models for those three variables, as well as multivariate models for potential combinations of two to three of these variables, were fitted. We found *n* of loose stools to be the most predictive. No meaningful adjusted *R*^2^ increase upon addition of another variable was seen in the multivariate models. Hence, only *n* of loose stools is included in MCS. The number of total stools a day is directly derived from the number of loose stools and the number of solid stools. The number of solid stools does not contribute meaningfully towards a diarrhea score, and the number of total stools is a worse predictor than the number of loose stools, which led to the exclusion of both variables from the MCS. Based on scores for the remaining items, MCS ranges from 0 (asymptomatic) to 15 (maximal symptoms; Table S2).

**Figure 5.**
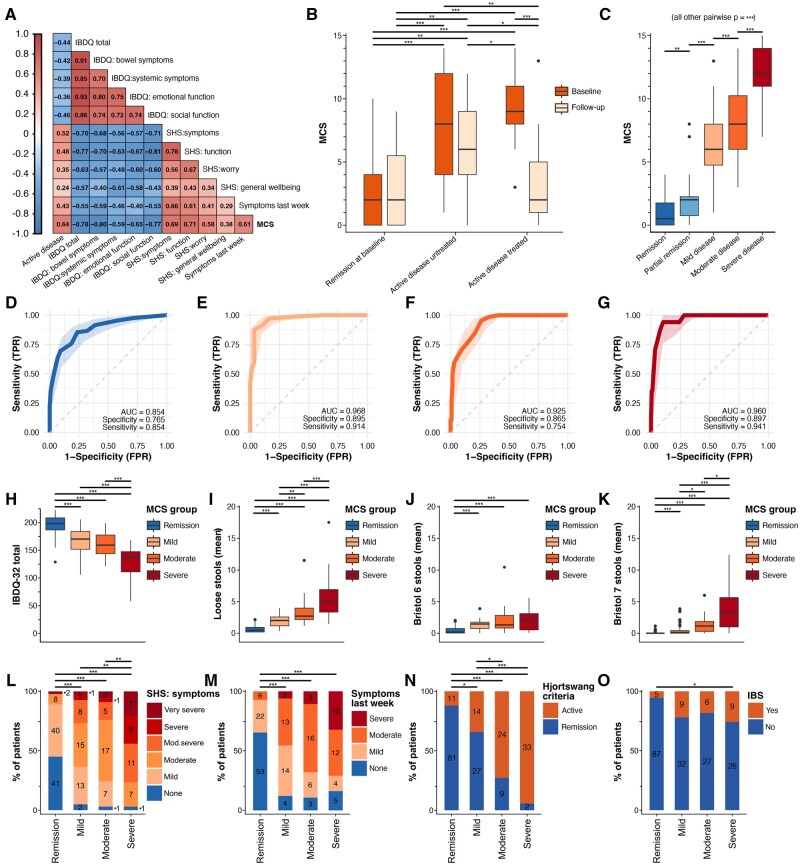
Validity, reliability, and responsiveness of the Microscopic Colitis Score (MCS). (A) Pearson coefficients for MCS correlation with disease activity, HRQoL, and patient experience of microscopic colitis symptoms. (B) Test–retest and responsiveness of MCS. Importantly, no significant difference was found between baseline and follow-up for patients (*n* = 42) in remission at baseline and those (*n* = 14) with untreated active disease, which supports good reliability. Responsiveness of MCS was supported by a substantial change in MCS for patients (*n* = 37) who were treated for active disease. (C) MCS values differed significantly between all previously identified clusters. To determine meaningful MCS cut-offs for disease severity grading, receiver operating characteristic (ROC) curves were calculated. Optimal MCS cut-off for (D) capturing active disease according to Hjortswang criteria, (E) active disease (mild, moderate, and severe) according to clustering, (F) moderate or severe disease according to clustering, and (G) severe disease according to clustering were determined. Optimal MCS cut-offs were MCS ≥ 4, MCS ≥ 4, MCS ≥ 7, and MCS ≥ 10 for (D)–(G) respectively. We used these MCS cut-offs to define severity groups (MCS groups): remission (MCS of 0–3), mild (4–6), moderate (7–9), severe (10–15). We visualize (H) IBDQ-32 total, (I) *n* of loose stools (mean), (J) *n* of Bristol 6 and (K) *n* of Bristol 7 stools (mean), (L) SHS symptoms, (M) patient-rated MC symptoms, (N) Hjortswang criteria, and (O) IBS co-morbidity by MCS groups (*x*-axis). Numbers in (L)–(O) indicate the number of patients corresponding to percentages on the *y*-axis. Significance bars were derived by pairwise Wilcoxon signed-rank tests (paired when applicable) and χ^2^ tests. All *P*-values were Bonferroni corrected. *P < .05; **P < .005; ***P < .0005; IBDQ, Inflammatory Bowel Disease Questionnaire; SHS, Short Health Scale.

Similar to MCSQ-related analyses above, we find that MCS is a valid, reliable, and responsive measure of microscopic colitis activity (Figure 5A–C; Table 3). In univariate and multivariate linear regression models, MCS yielded a strong level of explanatory power for HRQoL-related predictions, as illustrated for IBDQ-32 total (Table 3). Additionally, we found that MCS can distinguish between the MC severity groups previously identified by clustering (Figure 5C). Using ROC curves, we determined MCS cut-offs that resemble clustering-based disease severity and disease activity as defined by the Hjortswang criteria (Figure 5D–G). Such cut-offs aim to enhance clinical interpretability. Groups resulting from MCS cut-offs (remission; mild, moderate, and severe disease) correlated well with IBDQ-32 total (Pearson *r* = −0.73, *P* < 10^−16^; Figure 5H) and dimensions, as well as SHS symptoms (Spearman *r_s_* = 0.71, *P* < 10^−16^; Figure 5L) and SHS function (Spearman *r_s_* = 0.68, *P* < 10^−16^; Figure 5M).

**Table 3.** MCS in univariate and backward-eliminated multivariate linear regression for IBDQ-32 total.

	**Univariate model** *		
Variables	β	95% CI	*P*-value
(Intercept)	202.3	197.5 to 207.1	10^−139^
MCS	−6.0	−6.8 to −5.3	10^−34^

	Multivariate model**		
Variables	β	95% CI	*P*-value
(Intercept)	217.0	201.5 to 232.5	10^−59^
MCS	−5.1	−5.8 to −4.3	10^−24^
IBS: yes	−27.3	−36.9 to −17.6	10^−6^
Diagnosis (CC)			
LC	−7.2	−13.6 to −0.8	0.02
MC UNS	−15.3	−36.7 to 5.3	0.14
Country (Hungary)			
Spain	−7.4	−24.1 to 9.3	0.38
Sweden	−16.2	−30.4 to −2.0	0.03
Time: follow-up	5.9	−0.8 to 12.5	0.08

* Adj. *R*^2^=0.60; *P* < 10^−16^,

** Adj. *R*^2^=0.65; *P* < 10^−16^.

Eliminated variables: age, sex, disease duration, marital status, smoking, coexisting disease, MCS×IBS interaction. Abbreviations: MCS, Microscopic Colitis Score; IBS, irritable bowel syndrom; CC, collagenous colitis; LC, lymphocytic colitis; MC UNS, microscopic colitis unspecified.

No difference in IBDQ-32 total (Figure 5H) was seen between mild and moderate MCS group patients. However, patients with moderate disease had more loose stools and more often fulfilled the Hjortswang criteria (Figure 5I and N). This increase in *n* of loose stools was driven by watery diarrhea (Bristol 7) rather than fluffy stools (Bristol 6; Figure 5J and K). An increased number of watery stools in the moderate MC group aligns with the typical symptomatology of MC and might suggest a more significant intestinal upset compared to the mild MCS group, which experienced predominantly Bristol 6 diarrhea. Although speculative, the increased number of watery stools in the moderate MC group aligns with the typical symptomatology of MC and may suggest a more inflammatory intestinal environment compared to the mild MCS group, which experienced predominantly Bristol 6 diarrhea.^37^

While IBS co-morbidity appears to be somewhat overrepresented in active MC, multiple regression analysis revealed that the MCS × IBS interaction term did not achieve statistical significance, suggesting that IBS symptoms do not significantly inflate MCS. To further examine this, we repeated the validation of MCSQ and MCS using only the 111 patients who had no IBS comorbidity, in order to investigate whether MCSQ or MCS were prone to symptom inflation in IBS patients. The exclusion of IBS patients rather than specific subgroup analysis was chosen due to the relatively limited number of patients with IBS co-morbidity (*n* = 20). The outcomes of repeated validation analyses were comparable, suggesting no major confounding by IBS co-morbidity (Supplementary results).

## 4. Discussion

PROs are essential tools for the systematic collection of patient-centered data and monitoring of treatment outcomes.^19^^,^^20^ Their pivotal role in the drug approval process by regulatory agencies, such as the FDA and EMA, underscores their importance.^2^^,^^19^^,^^20^ However, existing PROs for MC fail to meet these regulatory standards. To address this gap, we developed MCSQ and MCS to assess disease severity in MC. The MCSQ encompasses a comprehensive 7-day assessment of stool patterns, urgency, repeated toilet visits, fecal leakage, and abdominal pain, capturing variations in MC symptoms.^21^ Based on MCSQ, we developed MCS, a new PRO measure to evaluate overall disease severity. Our study demonstrates that MCSQ and MCS are valid, reliable, and responsive measures of disease activity in both CC and LC.

One of the key advantages of MCS is that its systematic, data-driven characterization of MC severity provides both patients and physicians with a clear and practical framework to assess symptoms on a standardized scale, ranging from remission to mild, moderate, or severe disease. This might help to contextualize symptoms within the broader spectrum of the disease, offering a tangible and actionable framework for clinicians and promoting clearer patient communication.^38^^,^^39^ In contrast to MCDAI, MCS’s week-long assessment period provides a more sensitive measure of symptom fluctuations and better aligns with patient-centered clinical goals.^3^

As no definition for disease severity levels in MC existed, the use of clustering analysis to define five distinct disease severity groups is a notable strength of this study.^2^ By avoiding predefined assumptions, the Leiden algorithm provided an unbiased framework for subgroup identification.^40^ Notably, no single MCSQ variable was sufficient to distinguish severity levels. Instead, our results suggest that MC severity levels are best conceptualized as a continuous increase in symptom burden rather than discrete symptom profiles. MCS reliably distinguishes these subgroups of MC, providing a robust and practical tool for clinical and research applications.

While we do not find that IBS comorbidity majorly impacts MCS, the overrepresentation of IBS patients in the severe disease group should be considered a limitation, given the overlapping symptomatology. Such an overrepresentation of IBS is a limitation common to most studies regarding MC.^41^^,^^42^ Furthermore, an important limitation is that patients with active disease filled in MCSQs retrospectively; while this enables clinical useability, retrospective symptom reporting may introduce recall bias which in theory could enhance MCS responsiveness (treated active disease) and inflate test–retest reliability (untreated active disease). Additionally, the reliance on HRQoL measures for validation, while meaningful for capturing the effects of disease on patients’ daily lives, introduces potential variance due to subjectivity, causal ambiguity, and cross-population variability. Despite this, MCS explained most of the variance in the included HRQoL measures. Predictiveness (*R*^2^) of MCS far exceeds that of similar scores predicting IBDQ-32 or other HRQoL measures.^43–45^ We saw no variations in the relationship between MCS and HRQoL across countries, age groups, or genders. Another limitation is the unrecorded number of patients declining participation at the individual centers. Given this study’s focus on evaluating the impact of MC symptoms on HRQoL rather than to explore HRQoL in the general population of MC patients, this might be of minor importance for the development of MCS.

Future studies should validate MCS in larger, longitudinal, and international cohorts, incorporating diverse populations to ensure generalizability. Further exploration of MCS’s applicability in patients with coexisting IBS, along with the inclusion of emerging biomarkers for MC severity, could refine its clinical utility. Studies evaluating its use in monitoring long-term disease progression and therapeutic outcomes will further establish its role in clinical and research settings.

## 5. Conclusions

The development of the MCSQ and MCS addresses the critical need for a standardized, patient-reported measure of disease severity in MC. MCSQ and MCS demonstrated strong validity, reliability, and responsiveness, providing a robust framework for quantifying disease severity. Unlike existing tools, MCS offers improved granularity, aligns with FDA and EMA regulatory standards, and captures rapid symptom changes, enhancing its utility in both clinical practice and research.

## Supplementary Material

jjaf153_Supplementary_Data

## Data Availability

The data underlying this article cannot be shared publicly due to privacy of individuals who participated in the study. The data will be shared on reasonable request to the corresponding author.

## References

[1] Langner C , Aust D, Ensari A, et al; Working Group of Digestive Diseases of the European Society of Pathology (ESP) and the European Microscopic Colitis Group (EMCG). Histology of microscopic colitis-review with a practical approach for pathologists. Histopathology. 2015;66:613-626. 10.1111/his.12592 [published Online First: 2014/11/11] 25381724

[2] Miehlke S , Guagnozzi D, Zabana Y, et al European guidelines on microscopic colitis: United European Gastroenterology and European Microscopic Colitis Group statements and recommendations. United European Gastroenterol J. 2021;9:13-37. 10.1177/2050640620951905 [published Online First: 2021/02/24] PMC8259259 33619914

[3] Cotter TG , Binder M, Loftus EV, et al Development of a Microscopic Colitis Disease Activity Index: a prospective cohort study. Gut. 2018;67:441-446. 10.1136/gutjnl-2016-313051 27965284

[4] Bjornbak C , Engel PJ, Nielsen PL, et al Microscopic colitis: clinical findings, topography and persistence of histopathological subgroups. Aliment Pharmacol Ther. 2011;34:1225-1234. 10.1111/j.1365-2036.2011.04865.x[published Online First: 20111003] 21967618

[5] Miehlke S , Verhaegh B, Tontini GE, et al Microscopic colitis: pathophysiology and clinical management. Lancet Gastroenterol Hepatol. 2019;4:305-314. 10.1016/s2468-1253(19)30048-2[published Online First: 2019/03/13] 30860066

[6] Hjortswang H , Tysk C, Bohr J, et al Health-related quality of life is impaired in active collagenous colitis. Dig Liver Dis. 2011;43:102-109. 10.1016/j.dld.2010.06.004[published Online First: 2010/07/20] 20638918

[7] Nyhlin N , Wickbom A, Montgomery SM, et al Long‐term prognosis of clinical symptoms and health‐related quality of life in microscopic colitis: a case–control study. Aliment Pharmacol Ther. 2014;39:963-972. 24612051 10.1111/apt.12685

[8] Bohr J , Wickbom A, Hegedus A, et al Diagnosis and management of microscopic colitis: current perspectives. Clin Exp Gastroenterol. 2014;7:273-284. 10.2147/ceg.S63905[published Online First: 2014/08/30] 25170275 PMC4144984

[9] Hjortswang H , Tysk C, Bohr J, et al Defining clinical criteria for clinical remission and disease activity in collagenous colitis. Inflamm Bowel Dis. 2009;15:1875-1881. 19504614 10.1002/ibd.20977

[10] Chande N , Al Yatama N, Bhanji T, et al Interventions for treating lymphocytic colitis. Cochrane Database Syst Rev. 2017;7:Cd006096. 10.1002/14651858.CD006096.pub4[published Online First: 2017/07/14] 28702956 PMC6483541

[11] Kafil TS , Nguyen TM, Patton PH, et al Interventions for treating collagenous colitis. Cochrane Database Syst Rev. 2017;11:Cd003575. 10.1002/14651858.CD003575.pub6[published Online First: 20171111] 29127772 PMC6486307

[12] Sebastian S , Wilhelm A, Jessica L, et al Budesonide treatment for microscopic colitis: systematic review and meta-analysis. Eur J Gastroenterol Hepatol. 2019;31:919-927. 10.1097/meg.0000000000001456[published Online First: 2019/06/19] 31211724

[13] El Hage Chehade N , Ghoneim S, Shah S, et al Efficacy and safety of vedolizumab and tumor necrosis factor inhibitors in the treatment of steroid-refractory microscopic colitis: a systematic review and meta-analysis. J Clin Gastroenterol. 2024;58:789-799. 10.1097/mcg.0000000000001914[published Online First: 2023/09/05] 37668427

[14] Miehlke S , Madisch A, Kupcinskas L, et al; BUC-60/COC Study Group. Budesonide is more effective than mesalamine or placebo in short-term treatment of collagenous colitis. Gastroenterology. 2014;146:1222-1230.e2. 10.1053/j.gastro.2014.01.019 24440672

[15] Miehlke S , Aust D, Mihaly E, et al; BUG-1/LMC Study Group. Efficacy and safety of budesonide, vs mesalazine or placebo, as induction therapy for lymphocytic colitis. Gastroenterology. 2018;155:1795-1804.e3. 10.1053/j.gastro.2018.08.042 [published Online First: 2018/09/10] 30195447

[16] Münch A , Bohr J, Miehlke S, et al; BUC-63 investigators. Low-dose budesonide for maintenance of clinical remission in collagenous colitis: a randomised, placebo-controlled, 12-month trial. Gut. 2016;65:47-56. 10.1136/gutjnl-2014-308363 25425655 PMC4717436

[17] Best WR , Becktel JM, Singleton JW, et al Development of a Crohn’s disease activity index: national cooperative Crohn’s disease study. Gastroenterology. 1976;70:439-444. 1248701

[18] Minsk A , Cohen D. FDA issues final guidance on patient-reported outcome measures used to support labeling claims. Silver Spring: US Department of Health and Human Services.2010

[19] EMA. Reflection paper on the regulatory guidance for the use of health related quality of life (HRQL) measures in the evaluation of medicinal products. EMA. 2006

[20] U.S. Department of Health and Human Services FDA Center for Drug Evaluation and Research, U.S. Department of Health and Human Services FDA Center for Biologics Evaluation and Research, U.S. Department of Health and Human Services FDA Center for Devices and Radiological Health. Guidance for industry: patient-reported outcome measures: use in medical product development to support labeling claims: draft guidance. Health Qual Life Outcomes. 2006;4:79. 10.1186/1477-7525-4-79 [published Online First: 2006/10/13] 17034633 PMC1629006

[21] Lesnovska KP , Münch A, Bonderup O, et al The process of developing a disease activity index in microscopic colitis. J Crohns Colitis. 2022;16:452-459. 10.1093/ecco-jcc/jjab170 34562005

[22] Guyatt G , Mitchell A, Irvine EJ, et al A new measure of health status for clinical trials in inflammatory bowel disease. Gastroenterology. 1989;96:804-810. 10.1016/S0016-5085(89)80080-0 2644154

[23] Stjernman H , Grännö C, Järnerot G, et al Short health scale: a valid, reliable, and responsive instrument for subjective health assessment in Crohn’s disease. Inflamm Bowel Dis. 2008;14:47-52. 17828783 10.1002/ibd.20255

[24] Hjortswang H , Järnerot G, Curman B, et al The Short Health Scale: a valid measure of subjective health in ulcerative colitis. Scand J Gastroenterol. 2006;41:1196-1203. 16990205 10.1080/00365520600610618

[25] Lewis SJ , Heaton KW. Stool form scale as a useful guide to intestinal transit time. Scand J Gastroenterol. 1997;32:920-924. 10.3109/00365529709011203[published Online First: 1997/09/23] 9299672

[26] Beaton DE , Bombardier C, Guillemin F, et al Guidelines for the process of cross-cultural adaptation of self-report measures. Spine (Phila Pa 1976). 2000;25:3186-3191. 10.1097/00007632-200012150-00014[published Online First: 2000/12/22] 11124735

[27] Boeije H , Willis G. The Cognitive Interviewing Reporting Framework (CIRF). Methodology. 2013;9:87-95. 10.1027/1614-2241/a000075

[28] Solberg IC , Lygren I, Jahnsen J, et al; IBSEN Study Group. Clinical course during the first 10 years of ulcerative colitis: results from a population-based inception cohort (IBSEN Study). Scand J Gastroenterol. 2009;44:431-440. 10.1080/00365520802600961 19101844

[29] Palsson OS , Whitehead WE, van Tilburg MAL, et al Development and validation of the Rome IV Diagnostic Questionnaire for adults. Gastroenterology. 2016;150:1481-1491. 10.1053/j.gastro.2016.02.014

[30] Revelle W. psych: Procedures for psychological, psychometric, and personality research. R package version. 2020;2.

[31] Shrout PE , Fleiss JL. Intraclass correlations: uses in assessing rater reliability. Psychol Bull. 1979;86:420-428. 18839484 10.1037//0033-2909.86.2.420

[32] Koo TK , Li MY. A guideline of selecting and reporting intraclass correlation coefficients for reliability research. J Chiropr Med. 2016;15:155-163. 10.1016/j.jcm.2016.02.012[published Online First: 2016/06/23] 27330520 PMC4913118

[33] Butler A , Hoffman P, Smibert P, et al Integrating single-cell transcriptomic data across different conditions, technologies, and species. Nat Biotechnol. 2018;36:411-420. 10.1038/nbt.4096 29608179 PMC6700744

[34] Hlavaty T , Persoons P, Vermeire S, et al Evaluation of short-term responsiveness and cutoff values of inflammatory bowel disease questionnaire in Crohn’s disease. Inflamm Bowel Dis. 2006;12:199-204. 10.1097/01.Mib.0000217768.75519.32 16534421

[35] Child CG , Turcotte JG. Surgery and portal hypertension. Major Probl Clin Surg. 1964;1:1-85.[published Online First: 1964/01/01] 4950264

[36] Pugh RNH , Murray-Lyon IM, Dawson JL, et al Transection of the oesophagus for bleeding oesophageal varices. Br J Surg. 1973;60:646-649. 10.1002/bjs.1800600817 4541913

[37] Anbazhagan AN , Priyamvada S, Alrefai WA, et al Pathophysiology of IBD associated diarrhea. Tissue Barriers. 2018;6:e1463897. 10.1080/21688370.2018.1463897[published Online First: 2018/05/09] 29737913 PMC6179131

[38] Greenhalgh J , Gooding K, Gibbons E, et al How do patient reported outcome measures (PROMs) support clinician-patient communication and patient care? A realist synthesis. J Patient Rep Outcomes. 2018;2:42. 10.1186/s41687-018-0061-6 30294712 PMC6153194

[39] Ishaque S , Karnon J, Chen G, et al A systematic review of randomised controlled trials evaluating the use of patient-reported outcome measures (PROMs). Qual Life Res. 2019;28:567-592. 10.1007/s11136-018-2016-z 30284183

[40] Traag VA , Waltman L, van Eck NJ. From Louvain to Leiden: guaranteeing well-connected communities. Sci Rep. 2019;9:5233. 10.1038/s41598-019-41695-z[published Online First: 2019/03/28] 30914743 PMC6435756

[41] Kane JS , Irvine AJ, Derwa Y, et al High prevalence of irritable bowel syndrome-type symptoms in microscopic colitis: implications for treatment. Therap Adv Gastroenterol. 2018;11:1756284818783600. 10.1177/1756284818783600 PMC6024332 29977339

[42] Guagnozzi D , Arias Á, Lucendo AJ. Systematic review with meta-analysis: diagnostic overlap of microscopic colitis and functional bowel disorders. Aliment Pharmacol Ther. 2016;43:851-862. 10.1111/apt.13573[published Online First: 2016/02/26] 26913568

[43] Malik BA , Gibbons K, Spady D, et al Health-related quality of life in pediatric ulcerative colitis patients on conventional medical treatment compared to those after restorative proctocolectomy. Int J Colorectal Dis. 2013;28:325-333. 10.1007/s00384-012-1561-0 22914964

[44] González-Moret R , Cebolla-Martí A, Almodóvar-Fernández I, et al Inflammatory biomarkers and psychological variables to assess quality of life in patients with inflammatory bowel disease: a cross-sectional study. Ann Med. 2024;56:2357738. 10.1080/07853890.2024.2357738[published Online First: 2024/05/31] 38819080 PMC11146243

[45] Takahashi M , Nunotani M, Aoyama N. Construction of an explanatory model for quality of life in outpatients with ulcerative colitis. Inflamm Intest Dis. 2023;8:23-33. 10.1159/000530455[published Online First: 2023/07/05] 37404382 PMC10315687

