# Supplementary material for: Development and validation of a novel patient-reported outcome for microscopic colitis—Microscopic Colitis Score (MCS)
Source: J Crohns Colitis. 2025 Oct 13;19(9):jjaf153. doi: 10.1093/ecco-jcc/jjaf153 (PMC12527458; doi:10.1093/ecco-jcc/jjaf153)
Supplement: jjaf153_Supplementary_Data [file jjaf153_supplementary_data.zip › OP-ECCO250041_AuthorCorr_CmtAttachmentsFolder_Supplement submitted (1).pdf]

Development and Validation of a Novel patient-reported Outcome for Microscopic Colitis – Microscopic Colitis Score (MCS)

Katarina Pihl Lesnovska[1,*], RN PhD; Samuel Schäfer[1,2,*], MD PhD; Yamile Zabana[3,4], MD PhD; Ingrid Fajardo Anes[3], MD; Danila Guagnozzi[4,5], MD; Emese Mihaly[6], MD PhD; Stephan Miehlke[7], MD Professor; Ahmed Madisch[8], MD Professor; Beatrice Marinoni[9], MD; Giovanni Latella[10], MD Professor; Andreas Münch[1], MD PhD; Henrik Hjortswang[1], MD PhD

An initiative of the European Microscopic Colitis Group (EMCG).

[*] These authors contributed equally to this work.
[1] Department of Gastroenterology and Hepatology, Linköping University Hospital, Linköping and Department of Health, Medicine and Caring Sciences, Linköping University, Linköping, Sweden
[2] Department of Biomedical and Clinical Sciences, Linköping University, Linköping, Sweden
[3] Department of Gastroenterology, Hospital Universitari Mútua Terrassa, Terrassa, Spain
[4] Centro de Investigación Biomédica en Red en Enfermedades Hepáticas y Digestivas, Madrid, Spain
[5] Department of Gastroenterology, University Hospital Vall de Hebron, Barcelona, Spain
[6] Department of Internal Medicine, Semmelweis University, Budapest, Hungary
[7] Center for Digestive Diseases, Internal Medicine Center, Hamburg, Germany
[8] Center of Internal Medicine, Hospital DIAKOVERE Friederikenstift, Hannover, Germany.
[9] Gastroenterology and Endoscopy Unit, Fondazione IRCCS Ca' Granda Ospedale Maggiore Policlinico, University of Milan, Milan, Italy
[10] Gastroenterology, Hepatology and Nutrition Division, Department of Life, Health and Environmental Sciences, University of L'Aquila, L'Aquila, Italy.

Correspondence to Dr Samuel Schäfer; samuel.schafer@liu.se; Department of Gastroenterology and Hepatology, Linköping University Hospital, Linköping, and Department of Health, Medicine and Caring Sciences, Linköping University, Linköping, Sweden.

Supplementary results

Validation of MCSQ and MCS in patients with no co-existing IBS

We repeated the validation of MCSQ and MCS using only 111 patients with no co-existing IBS to demonstrate that symptom burden in MCSQ and MCS scores were not systematically inflated by the presence of IBS patients in our cohort.

MCSQ

Validity was again explored using EFA. Eligibility of data for was confirmed by Bartlett's test of sphericity ($p < 10^{-300}$) and KMO of 0.700. Again, a three-factor-model was derived. Item loading for the three-factor model ranged from 0.37 (mean n of stools) to 0.98 (mean n of loose stools). Promax rotation of factors in the three-factor model produced comparable intercorrelations to **Figure 1c**. Cronbach's alpha (coefficient alpha reliability) was 0.891 for Factor 1 loadings (>|0.4|) and 0.832 for all items in MCSQ. We, again, found that all MCSQ items exhibited strong correlations (>|0.3|) to relevant measures (**Supplementary Figure 2a**).

Intraclass correlation coefficients were re-calculated using only derived from comparing responses of patients between baseline and follow-up for patients that were in remission at both time points. Overall ICC (95% CI) for MCSQ was 0.93 (0.89-0.95) corresponding to excellent reliability. Similarly, we assessed ICC for 11 patients that have had active disease at baseline but received no treatment and had still active disease at follow-up two weeks later. Overall ICC was 0.92 (0.89-0.95).

Paired analysis of patients that had active disease at baseline and were treated showed a significant improvement in all MCSQ items comparable to that seen for all patients (including IBS patients; **Figure 2c**).

MCS

We also repeated analyses concerning MCS validity, reliability and responsiveness now excluding all IBS patients. We find that MCS continues to be a valid, reliable and responsive measure of microscopic colitis activity (**Supplementary Figure 2, Supplementary Table 3**). The univariate and multivariate linear regression models for predicting IBDQ-32 total using MCS of non-IBS patients were comparable to regression that included all patients (**Supplementary Table 3**). Regressions still indicated MCS to have a strong predictive value. Similarly, we reassured that the suggested MCS cut-offs with a high sensitivity and specificity could distinguish between clustering-based disease severity groups and disease activity as defined by the Hjortswang criteria (**Supplementary Figure 2d-g**).

Collectively, we conclude that MCSQ and MCS performs similarly in patients with and without IBS. Though, our analysis indicates that patients with IBS co-morbidity have an independently decreased HRQoL (**Table 3**).

Supplementary Table 1. Eigenvalues and total variance for three-factor MCSQ model.

	Initial Eigenvalues			Extracted, unrotated factors			Promax rotated factors		
**Component**	**Total**	**% of Variance**	**Cum. %**	**Total**	**% of Variance**	**Cum. %**	**Total**	**% of Variance**	**Cum. %**
1	5·0	51	51	4·7	46	46	4·0	40	40
2	1·1	11	62	1·0	10	56	1·7	17	57
3	1·0	10	73	1·0	10	66	1·2	12	69
4	0·8	8	81						
5	0·7	7	88						
6	0·5	6	93						
7	0·4	4	97						
8	0·3	3	100						
9	0·0	0	100						
10	0·0	0	100						

Abbreviations Cum., cumulative % of variance.

Supplementary Table 2. MCS scoring

Variables \ MCS points	0p	1p	2p	3p
Mean n nocturnal stools per day	<0.14	≥0.14	≥0.56	-
Mean n loose stool per day	<1	≥1	≥2	≥3
Need for repeated stool	<0.14	≥0.14	≥0.56	-
Urgency	<0.28	≥0.28	≥1	≥2
Leakage	<0.28	-	≥0.28	-
Abdominal pain	<0.56	≥ 0.56	≥1.28	≥2

MCS can range from 0p to 15p. We suggest that disease severity might be classified as follows: 0-3p symptomatic remission; 4-6p mild disease; 7-9p moderate disease; 10-15p severe disease.

Supplementary Table 3. MCS (of non-IBS patients) in univariate and backward-eliminated multivariate linear regression for IBDQ-32 total.

	Univariate model*		
**Variables**	**β**	**95% CI**	**p-value**
(Intercept)	202.4	197.9 to 206.9	10^{-130}
MCS	-5.5	-6.2 to -4.7	10^{-29}
	**Multivariate model****		
**Variables**	**β**	**95% CI**	**p-value**
(Intercept)	198.1	184.5 to 211.7	10^{-56}
MCS	-4.7	-5.5 to -3.9	10^{-21}
Country (Hungary)			
Spain	11.9	-5.1 to 28.9	0.17
Sweden	-3.1	-16.0 to 9.8	0.64
Time: follow-up	8.4	1.8 to 15.0	0.01

* Adj. $R^2 = 0.61$. $p < 10^{-15}$. ** Adj. $R^2 = 0.63$. $p < 10^{-15}$. Eliminated variables: age, sex, diagnosis, disease duration, marital status, smoking, coexisting disease.

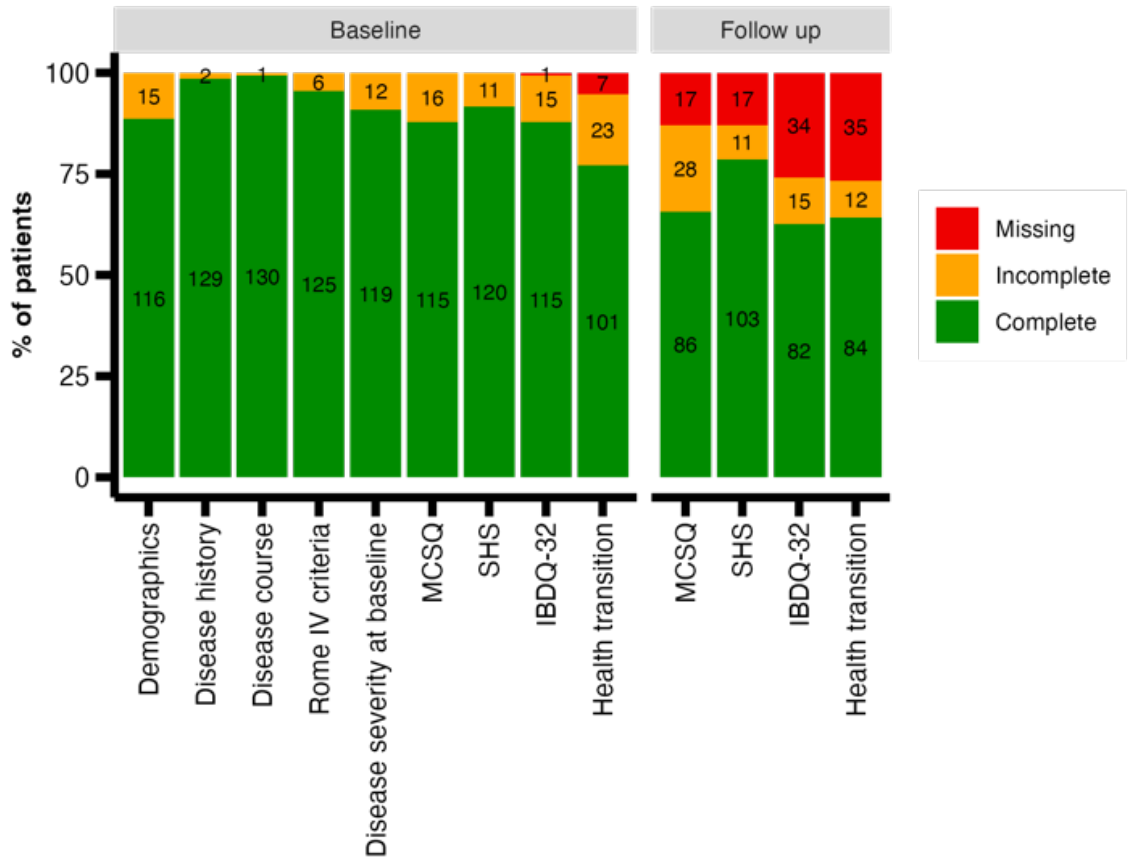

Supplementary Figure 1. Questionnaire completion at baseline and follow-up. Partially completed questionnaires were used in analysis whenever feasible to minimize data loss.

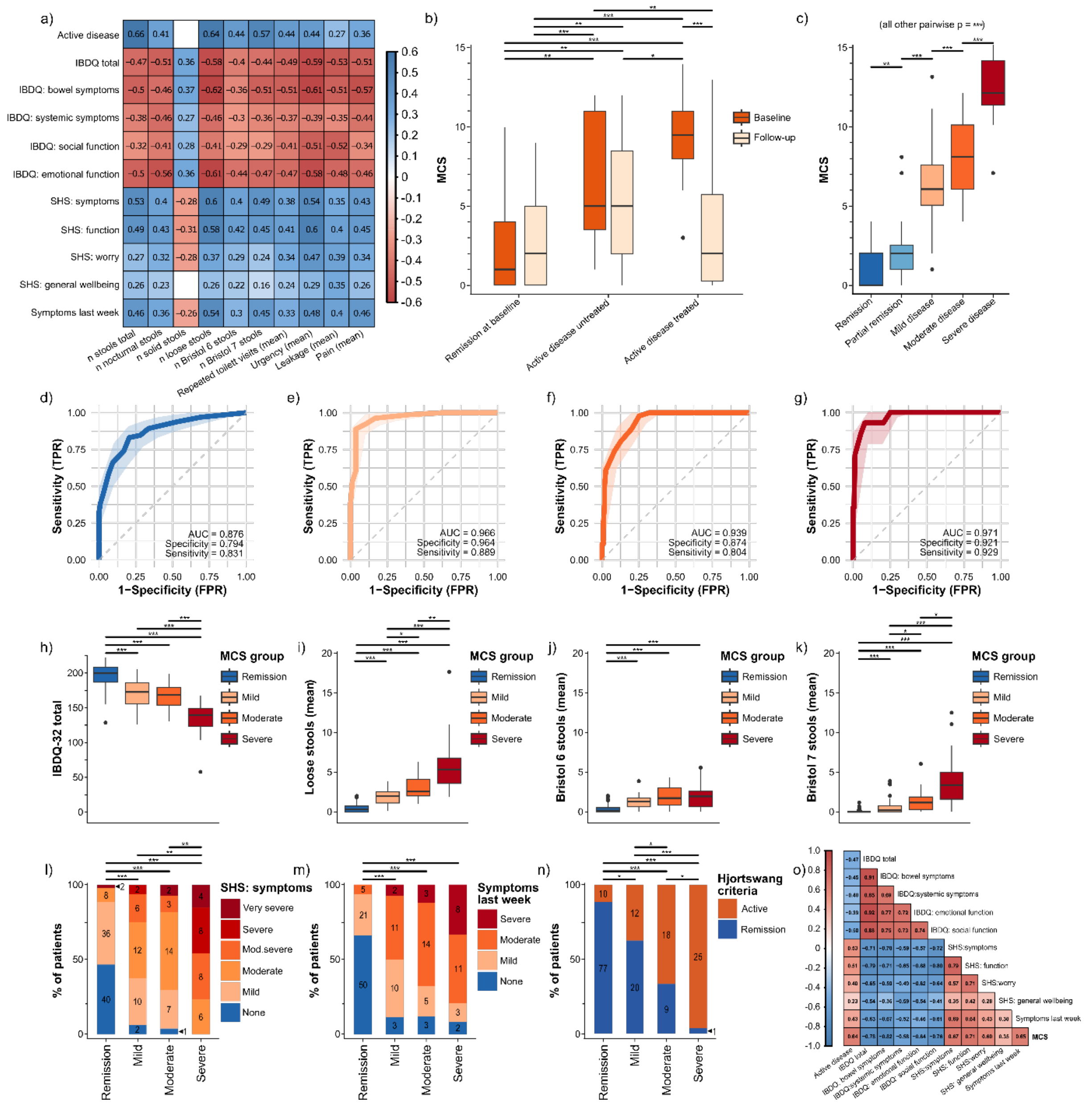

Supplementary Figure 2. Validity, reliability and responsiveness of the Microscopic Colitis Score (MCS) for non-IBS patients. a) Pearson coefficients for MCSQ item's correlation with disease activity, HRQoL and patient experience of microscopic colitis symptoms. Active disease (0=remission, 1=active disease) as per Hjortswang criteria. The 6-point Likert scale for SHS ranged from 1 (best) to 6 (worst). Patients rated the occurrence of microscopic colitis symptoms during the previous week ranged from 0 (none) to 3 (severe). b) Test-retest and responsiveness of MCS. Importantly, no significant difference was found between baseline and follow-up for patients in remission at baseline and patients with untreated active disease, which supports good reliability. Responsiveness of MCS was supported by a substantial change in MCS for patients that were treated for active disease. c) MCS values differed significantly between all clusters. To determine meaningful MCS cut-offs for disease severity grading, ROC curves were calculated. MCS cut-offs with optimal sensitivity and specificity were determined for d) capturing active disease according to Hjortswang criteria, e) active disease (mild, moderate and severe) according to clustering, f) moderate or severe

disease according to clustering and g) severe disease according to clustering were determined. Optimal MCS cut-offs were MCS ≥ 4, ≥ 4, ≥ 7 and ≥ 10 for d-e) respectively which corresponded to earlier identified cut-offs using all patients (including IBS patients). Using the MCS cut-offs to define severity groups, we visualized h) IBDQ-32 total, i) n of loose stools (mean), j) n of Bristol 6 and k) Bristol 7 stools (mean), l) SHS symptoms, m) patient-rated MC symptoms, and n) Hjortswang criteria. Numbers in l-n) indicate n of patients complementary to percentages on the y-axis. o) Pearson coefficients for MCS correlation with disease activity, HRQoL and patient experience of microscopic colitis symptoms shows a considerable correlation ($|r| \geq 0.6$) to most HRQoL measures indicating validity. Significance bars were derived by pairwise Wilcoxon signed-rank tests (paired when applicable) and Chi2 tests. All p-values were Bonferroni corrected. Abbreviations: *, $p < 0{\cdot}05$; **, $p < 0{\cdot}005$; ***, $p < 0{\cdot}0005$.
